# Association between Polymorphism of Genes *IL-1A*, *NFKB1*, *PAR1*, *TP53,* and *UCP2* and Susceptibility to Non-Small Cell Lung Cancer in the Brazilian Amazon

**DOI:** 10.3390/genes14020461

**Published:** 2023-02-10

**Authors:** Esdras E. B. Pereira, Antônio A. C. Modesto, Bruno M. Fernandes, Rommel M. R. Burbano, Paulo P. Assumpção, Marianne R. Fernandes, João F. Guerreiro, Sidney E. B. dos Santos, Ney P. C. dos Santos

**Affiliations:** 1 Laboratory of Human and Medical Genetics, Institute of Biological Science, Federal University of Pará, Belem 66077-830, PA, Brazil; 2 Oncology Research Center, Federal University of Pará, Belem 66073-005, PA, Brazil; 3 Instituto Tocantinense Presidente Antônio Carlos (ITPAC), Abaetetuba 68440-000, PA, Brazil

**Keywords:** genetic polymorphism, biomarker, non-small cell lung cancer, susceptibility

## Abstract

Non-small cell lung cancer (NSCLC) accounts for the vast majority of cases of lung neoplasms. It is formed in multiple stages, with interactions between environmental risk factors and individual genetic susceptibility and with genes involved in the immune and inflammatory response paths, cell or genome stability, and metabolism, among others. Our objective was to evaluate the association between five genetic variants (*IL-1A*, *NFKB1*, *PAR1*, *TP53,* and *UCP2*) and the development of NSCLC in the Brazilian Amazon. The study included 263 individuals with and without lung cancer. The samples were analyzed for the genetic variants of *NFKB1* (rs28362491), *PAR1* (rs11267092), *TP53* (rs17878362), *IL-1A* (rs3783553), and *UCP2* (INDEL 45-bp), which were genotyped in PCR, followed by an analysis of the fragments, in which we applied a previously developed set of informative ancestral markers. We used a logistic regression model to identify differences in the allele and the genotypic frequencies among individuals and their association with NSCLC. The variables of gender, age, and smoking were controlled in the multivariate analysis to prevent confusion by association. The individuals that were homozygous for the Del/Del of polymorphism *NFKB1* (rs28362491) (*p* = 0.018; OR = 0.332) demonstrate a significant association with NSCLC, which was similar to that observed in the variants of *PAR1* (rs11267092) (*p* = 0.023; OR = 0.471) and *TP53* (rs17878362) (*p* = 0.041; OR = 0.510). Moreover, the individuals with the Ins/Ins genotype of polymorphism *IL-1A* (rs3783553) demonstrated greater risk for NSCLC (*p* = 0.033; OR = 2.002), as did the volunteers with the Del/Del of *UCP2* (INDEL 45-bp) (*p* = 0.031; OR = 2.031). The five polymorphisms investigated can contribute towards NSCLC susceptibility in the population of the Brazilian Amazon.

## 1. Introduction

Lung neoplasm is among the prevailing cancer types in the world, representing a little over 11% of all neoplasms and responsible for 18% of the deaths by the disease [1]. Non-small cell lung cancer (NSCLC) represents up to 85% of that neoplasm [2,3]. In Brazil, that prevalence may reach 90%, and most cases are detected in the advanced stages of the disease [4,5].

The formation of lung neoplasms may occur in several stages, with synergistic and complex interactions between the environmental risk factors, such as smoking and individual genetic susceptibility [6]. That susceptibility is associated with genetic polymorphisms, which include genes involved in the metabolism, the activation of carcinogens in tobacco smoke, DNA repair, the regulation of the cell cycle, homeostasis, and the immune response, among other paths [7,8]. The alterations present in those genes may generate imbalances in those paths and trigger the development of various neoplasms [7,8,9].

In this study, five important genetic variants for immune response pathways, cell cycle control, homeostasis, angiogenesis, and metabolism were investigated: *IL-1A* (rs3783553), *NFKB1* (rs28362491), *TP53* (rs17878362), *PAR1* (rs11267092), and *UCP2* (INDEL 45-bp). These genes and their variants have been associated with other cancers, including leukemias and breast, colorectal, gastric, prostate, and head and neck cancers, in different populations [7,9,10,11,12,13,14,15,16,17,18].

The choice for those markers was based on the fact that they are potential influencers in the development of neoplasms. Thus, identifying the associations of those polymorphisms with NSCLC may enable the tracking of individuals with greater disease susceptibility, before the first symptoms, enabling monitoring and early treatment, which may reduce the morbidity and mortality from the neoplasm.

The aim of this study was to evaluate the association between the *IL-1A* (rs3783553), *NFKB1* (rs28362491), *TP53* (rs17878362), *PAR1* (rs11267092), and *UCP2* (INDEL 45-bp) susceptibilities to NSCLC in the Brazilian Amazon.

## 2. Materials and Methods

### 2.1. Ethical Compliance

It is an observational, case-control study, authorized by the research Ethics Committees of the Oncology Research Center, under the CAAE protocol number: 37386214.3.0000.5634, and by the João de Barros Barreto University Hospital, under CAAE protocol number: 37386214.3.3001.0017, both in the city of Belém-Pará, within the Amazon region of Brazil. All the individuals signed an informed consent form.

### 2.2. Case and Control

The volunteers were recruited in public health centers, from both genders, with no family ties to each other, and from the same socio-economic level. The data and samples from 263 individuals were collected, whereas 67 patients had NSCLC (case group), as defined and classified in the histopathological exam, and 196 patients had no type of cancer (control group). The groups had demographic and clinical data collected, which included age, gender, and smoking history.

### 2.3. DNA Extraction and Quantification

The extraction of the leukocytes’ genomic DNA from the peripheral blood was executed using a Mini Spin Plus Kit (P. 250, Biopur, Biometrix, Gronsveld, the Netherlands) according to the manufacturer’s recommendations. The DNA’s concentration and purity were measured with a NanoDrop 1000 spectrophotometer (Thermo Scientific NanoDrop 1000; NanoDrop Technologies, Wilmington, DE, USA).

### 2.4. Genotyping

The five genetic variants were genotyped by a multiplex PCR reaction followed by a capillary electrophoresis. The primers detailed in Table 1 were used for the amplifications. The analysis of the PCR amplicons was carried out based on an electrophoresis using the ABI Prism 3130 sequencer and the GeneMapper ID v.3.2 software [19]. The electrospherogram of the markers (*NFKB1*, *TP53*, *PAR1*, *UCP2*, and *IL-1A*) investigated and interpreted by GeneMapper ID v. 3.2 software, is exposed and detailed in Figure S1.

### 2.5. Analysis of the Hardy–Weinberg Equilibrium (HWE)

The allele and genotype frequency of the genetic variants was determined by a direct count of the alleles, followed by the calculation of the Hardy–Weinberg equilibrium (HWE) using the standard parameters of the Arlequin 3.5.1.2 software (Swiss Institute of Bioinformatics, Bern, Switzerland). All the genetic variants were shown to be present in the HWE (Table 2).

### 2.6. Genetic Ancestry Analysis

The genotyping was performed to analyze the ancestry of the samples; it was performed according to Ramos et al. [20], using 61 informative markers of autosomal ancestry in three PCR multiplex reactions. The amplicons were analyzed by electrophoresis using the ABI Prism 3130 sequencer and the GeneMapper ID v. 3.2 software (Applied Biosystems, Life Technologies, Carlsbad, CA, USA). The electrospherogram of the 61 markers of genomic ancestry, interpreted by GeneMapper ID v. 3.2 software, is exposed and detailed in Figure S2. The individual proportions of European, African, and Amerindian genetic ascendancy were estimated using the Structure v. 2.3.3 software (Stanford University, Stanford, CA, USA), assuming three parental populations [21].

### 2.7. Statistic Analysis

The statistical analyses were made using the SPSS 20.0 statistics package (IBM, Armonk, NY, USA). For the comparative analysis between the study groups with regard to the demographic and clinical variables, Pearson’s chi-square and the Mann–Whitney test were both applied. To analyze the association of the polymorphisms with a lung cancer risk, a logistics regression was made, estimating the odds ratio (OR) and the reliability intervals of 95% (IC). The variables of gender, age, and smoking were controlled in that multivariate analysis to prevent confusion by association. A significance level of *p* < 0.05 was considered for all statistical analyses. In addition, a multifactorial dimensionality reduction (MDR) analysis was performed using MDR 3.0.2 (Vanderbilt University Medical School, Nashville, TN, USA) to assess the SNP-SNP viability associated with NSCLC risk, as described in Supplementary Table S1 and Supplementary Figure S3.

## 3. Results

In the results related to the demographic and clinical aspects, we may observe that the groups differed as to gender, age, and smoking history. Most of the NSCLC patients were male with an average age of 60 years and had a smoking history (Table 3). The ancestry analysis performed revealed that the case and control groups had a similar ancestry genome profile, with a larger European contribution for both populations (Table 3).

The analysis of the genotype and allele distribution revealed that the polymorphisms *NFKB1* (rs28362491), *PAR1* (rs11267092), and *TP53* (rs17878362) presented variations associated with those at the lowest risk for the development of NSCLC, whereas the variations of the polymorphisms of gene *IL-1A* (rs3783553) and *UCP2* (INDEL 45-bp) were associated with the risk of developing the disease (Table 4).

For *NFKB1* (rs28362491), the genotype Del/Del also demonstrated an association with the NSCLC in comparison with the different genotypes (*p =* 0.018; OR *=* 0.332; 95% CI = 0.133–0.825). That is similar to what was observed for the polymorphisms PAR1 (rs11267092) and *TP53* (rs17878362), where their Del/Del genotypes also demonstrated an association with the lowest risk for the development of NSCLC (Table 4).

In contrast, the polymorphism of the *IL-1A* (rs3783553) gene presented its Ins/Ins genotype as being associated with a higher risk of developing NSCLC (*p =* 0.033; OR *=* 2.002; 95% CI: 1.059–3.546), which was similar to the allele Ins. Similar results were identified for the polymorphism of gene *UCP2* (INDEL 45-bp), where individuals with the Del/Del genotype had about twice the risk of developing the disease compared to the different genotypes (*p =* 0.031; OR *=* 2.031; 95% CI = 1.067–3.868) (Table 4).

## 4. Discussion

Lung neoplasm is the second class of cancer with the highest volume of detection in both genders globally, prevailing more among male individuals aged over 65 and frequently associated with smoking [1,22,23]. In the Brazilian population specifically, that neoplasm is the third most common among males and the fourth among females, whereas more than 8% of the cases are related to smoking [4,24]. That is also maintained for NSCLC, where a larger prevalence was observed among men over 60 [5,25,26]. In addition, that is similar to what was observed in this research, where the individuals with NSCLC were shown to be mostly men above 60 with a history of smoking.

The frequency of lung neoplasm among males is typically connected to tobacco consumption since the ratio of male smokers is larger than that of female smokers. Male individuals are also exposed to carcinogenic agents in some work activities, which favors a higher prevalence of the disease in that group [27,28,29]. However, those differences vary among countries according to their level of socio-economic and cultural development and are associated with tobacco consumption and exposure to both intrinsic and extrinsic risk factors [1,30].

In terms of age, we observe that at least one third of lung neoplasm cases are diagnosed between 65 and 74 years of age, whereas the elderly encompass nearly two thirds of all cases [1]. The aging process is associated with genomic modifications, favoring the accumulation of cells with the most varied molecular alterations, which compromise internal homeostasis, increasing individual susceptibility to carcinogens and, as a result, lung carcinogenesis [31,32].

In this study, upon assessing the individual susceptibility to the development of NSCLC, we may observe relevant results with respect to the polymorphisms of *NFKB1* (rs28362491), *PAR1* (rs11267092), and *TP53* (rs17878362), all three of which are associated with the reduction in the risk of developing NSCLC, whereas the polymorphisms of *IL-1A* (rs3783553) and *UCP2* (INDEL 45-bp) are associated with a higher risk of developing that neoplasm.

The *NFKB1* gene expresses the NFkB1 (p50/p105) transcription factor, activated by several intracellular and extracellular stimuli, favoring the repression of gene transcription [18,33]. In the polymorphism of *NFKB1* (rs28362491), it occurs from the *NFKB1* gene-promoting region and is related to a susceptibility to multiple diseases associated with inflammation, immunity, and tumorigenesis [34,35]. In our study, individuals homozygous for Del/Del of this genetic variant demonstrated an association effect against the development of NSCLC. Other studies have also identified associations of this polymorphism with lung cancer [36,37]. That is because the genotype is associated with lower transcriptional activity and a reduction in p50/p105 expression, acting as a counterpart to the tumorigenesis events [18,38].

The same was observed with the polymorphism of *PAR1* (rs11267092), where individuals with genotype Del/Del had less chances of developing NSCLC. The gene *PAR1* expresses the PAR1 receptor, which regulates the physiological processes of the cardiovascular, respiratory, and neurological systems and inflammation, embryogenesis, and carcinogenesis [20,39]. The *PAR1* (rs11267092) polymorphism occurs in the gene promoting region and modulates PAR1 production and activity, influencing many physiological events [40]. The reduction in mRNA expression by PAR1 correlates to the reduction in the invasive properties of some types of cancers [41,42]. In addition, it has been observed that the Del allele is associated with a better prognosis in some kinds of solid cancers [38,43], which corroborates the results of this study.

A similar outcome was observed in the polymorphism of *TP53* (rs17878362), where the individuals with the genotype Del/Del presented a lower risk for NSCLC. The gene *TP53* encodes the p53 protein, which is regulated by multiple mechanisms in response to a broad range of antiproliferative responses [9]. The polymorphism of *TP53* (rs17878362) is associated with many types of cancers, owing to the alterations in gene expression and protein function [9,16]. Corroborating our results, the studies show that the Del/Del genotype of the genetic variants is associated with higher levels of TP53 mRNA and a greater DNA repair capability than the alleles Ins/Del and Ins/Ins, granting more protection against carcinogenesis events [9,44,45].

In turn, the polymorphism of *IL-1A* (rs3783553) presented an association with the risk of NSCLC development. The individuals with the genotype Ins/Ins presented twice the susceptibility to neoplasm compared to the other genotypes. The gene *IL-1A* expresses interleukin IL-1A, a pro-inflammatory cytokine produced by monocytes and macrophages, released in response to cell injury, which may influence proliferation, angiogenesis, and tumor invasion, among other carcinogenic events [46]. Polymorphism rs3783553 is associated with the regulation of the IL-1A expression levels, for interrupting a linking site to miRNA-122 and miRNA-378, and is thus associated with several kinds of malignant neoplasms [10,47]. The studies indicate that the Ins/Ins genotype and the Ins allele of that polymorphism were also associated with a greater risk of developing cancer in general [10], corroborating our findings. 

In this study, the polymorphism in *UCP2* (INDEL 45-bp) also revealed an association with NSCLC susceptibility. The individuals with genotype Del/Del presented twice the risks for developing the disease. The gene *UCP2* expresses uncoupling protein 2 (UCP2), which acts as a proton transporter in mitochondria and is involved in energy homeostasis and thermogenesis, among other metabolic phenomena [48]. That gene and its variants have been associated with chronic diseases and some types of cancer [49,50]. Polymorphism *UCP2* (INDEL 45-bp) may alter the mRNA stability of UCP2 [51]. According to Esterbauer et al. [52], the half-life of mRNA from individuals with the deletion allele is greater than that from those with the insertion allele, which may indicate greater expression. That greater expression may influence carcinogenesis since the latter mitigates the production of reactive species of oxygen (ROS), protecting the neoplastic cell of the apoptotic path [53,54], which clarifies the results obtained in this study.

The MDR was used to analyze the interaction of these five SNPs. The results of the MDR model analysis of SNP-SNP showed no significant influence between the investigated polymorphisms. However the dendrogram and the Fruchterman–Reingold graph describe these interactions. The strongest interaction effect was found between PAR1 (rs11267092) and IL-1A (rs3783553), with information gain values of 2.11%, which were SNPs associated with risk reduction for NSCLC. Furthermore, the second strongest effect was found between TP53 (rs17878362) and NFKB1 (rs28362491), with information gain values of 0.70%, and between TP53 (rs17878362) and UCP2 (INDEL 45-bp), with information gain values of 0.50%, which were SNPs associated with an increased risk for NSCLC.

Our findings reinforce the results of other studies with other populations, which had a more expressive sample number but also observed a relationship between these genes and carcinogenesis. It is one of the few that investigated the association of these five variants with NSCLC, which is extremely important for the Amazon because it has an extremely mixed population; it is unique and little studied, and there is a lack of epidemiological and genetic information about the disease in the region. In the future, the use of this information as a screening tool will be able to identify individuals with greater susceptibility to NSCLC, favoring the establishment of preventive measures and early diagnosis and reducing the cost to health services and the morbidity and mortality rates of this neoplasm.

Importantly, this study is one of the few that has investigated the association of these five polymorphisms with NSCLC. Additional epidemiological investigations involving larger groups of individuals should be performed to confirm the results and determine whether these variants are isolated risk factors or associated with environmental factors, including smoking.

## 5. Conclusions

The five polymorphisms investigated show a significant association with NSCLC in the population of the Brazilian Amazon region. The genotypes of the polymorphisms of *NFKB1* (rs28362491), *PAR1* (rs11267092), and *TP53* (rs17878362) were associated with a lower risk for disease development, and the genotypes of the polymorphisms of *IL-1A* (rs3783553) and *UCP2* (INDEL 45-bp) were associated with a growth in the susceptibility to that malignant neoplasm.

## Figures and Tables

**Table 1:** Technical characteristics of the markers studied.

Gene	ID	Type	Length	Primers	Amplicon
*IL-1A*	rs3783553	INDEL	4 bp	F-5′TGGTCCAAGTTGTGCTTATCC3′	230–234 bp
				R-5′ACAGTGGTCTCATGGTTGTCA3′	
*NFKB1*	rs28362491	INDEL	4 bp	F-5′TATGGACCGCATGACTCTATCA3′	366–370 bp
				R-5′GGCTCTGGCATCCTAGCAG3′	
*PAR1*	rs11267092	INDEL	13 bp	F-5′AAAACTGAACTTTGCCGGTGT3′	265–277 bp
				R-5′GGGCCTAGAAGTCCAAATGAG3′	
*TP53*	rs17878362	INDEL	16 bp	F-5′GGGACTGACTTTCTGCTCTTGT3′	148–164 bp
				R-5′GGGACTGTAGATGGGTGAAAAG3′	
*UCP2*	*INDEL 45-bp*	INDEL	45 bp	F-5′CCCACACTGTCAAATGTCAACT3′	119–164 bp
				R-5′CCATGCTTTCCTTTTCTTCCT3′	

F: Forward; R: Reverse; INDEL: Insertion/Deletion.

**Table 2:** Hardy–Weinberg equilibrium of the investigated genetic variants.

Gene	ID	Genotype	Frequency	Hardy-Weinberg Equilibrium (*p*-Value)
*IL-1A*	rs3783553	Del/Del	37 (14.1%)	0.095
		Ins/Del	140 (53.2%)	
		Ins/Ins	86 (32.7%)	
*NFKB1*	rs28362491	Del/Del	59 (22.4%)	0.919
		Ins/Del	132 (50.2%)	
		Ins/Ins	72 (27.4%)	
*PAR1*	rs11267092	Del/Del	131 (49.8%)	0.644
		Ins/Del	107 (40.7%)	
		Ins/Ins	25 (9.5%)	
*TP53*	rs17878362	Del/Del	163 (62.0%)	0.575
		Ins/Del	90 (34.2%)	
		Ins/Ins	10 (3.8%)	
*UCP2*	*INDEL 45-bp*	Del/Del	127 (48.3%)	0.922
		Ins/Del	112 (42.6%)	
		Ins/Ins	24 (9.1%)	

Ins: Insertion; Del: Deletion.

**Table 3:** Demographic and clinical characteristics of the investigated groups.

Characteristics	Case (n = 67)	Control (n = 196)	*p*-Value
**Gender**			
Male	47 (70.1%)	62 (31.6%)	<0.001 ^a^*
Female	20 (29.9%)	134 (68.4%)	
**Ages (years)**			
Mean (±sd)	60.4 (±12.3)	70.5 (±8.2)	<0.001 ^b^*
**Smoking**			
Never smoked	13 (19.4%)	93 (47.4%)	<0.001 ^a^*
Smoker	54 (80.6%)	97 (49.5%)	
**Ancestry**			
European	47.1 (±16.9)	45.2 (±17.0)	0.521 ^b^
Amerindian	30.3 (±13.6)	30.6 (±14.8)	0.912 ^b^
African	22.6 (±12.1)	24.2 (±13.8)	0.498 ^b^

sd: standard deviation; ^a^. chi-square test; ^b^. Mann–Whitney test. *. *p*-value < 0.05.

**Table 4:** Genotypic and allelic distributions of investigated polymorphisms for non-small cell lung cancer patients compared to the control group.

Polymorphisms	Case (n = 67)	Control (n = 196)	*p*-Value ^a^	OR (95% CI)
***IL-1A* (*rs3783553*)**				
Del/Del	5 (7.5%)	32 (16.3%)	0.033 *	Ins/Ins vs. Others
Ins/Del	33 (49.3%)	107 (54.6%)		
Ins/Ins	29 (43.2%)	57 (29.1%)		2.002 (1.059–3.546)
Allele Del	43 (32.1%)	171 (43.6%)	0.033 *	0.499 (0.264–0.944)
Allele Ins	91 (67.9%)	221 (56.4%)		2.002 (1.059–3.546)
***NFKB1* (*rs28362491*)**				
Del/Del	13 (19.4%)	46 (23.5%)	0.018 *	Del/Del vs. Others
Ins/Del	35 (52.2%)	97 (49.5%)		
Ins/Ins	19 (28.4%)	53 (27.0%)		0.332 (0.133–0.825)
Allele Del	61 (45.5%)	189 (48.2%)	0.981	1.009 (0.500–2.033)
Allele Ins	73 (54.5%)	203 (51.8%)		0.992 (0.492–1.999)
***PAR1* (*rs11267092*)**				
Del/Del	23 (34.3%)	108 (55.1%)	0.023 *	Del/Del vs. Others
Ins/Del	32 (47.8%)	75 (38.3%)		0.471 (0.247–0.971)
Ins/Ins	12 (17.9%)	13 (6.6%)		
Allele Del	78 (58.2%)	291 (74.2%)	0.130	0.477 (0.183–1.244)
Allele Ins	56 (41.8%)	101 (25.8%)		
***TP53* (*rs17878362*)**				
Del/Del	34 (50.7%)	129 (65.8%)	0.041 *	Del/Del vs. Others
Ins/Del	29 (43.3%)	61 (31.1%)		0.510 (0.267–0.974)
Ins/Ins	4 (6.0%)	6 (3.1%)		
Allele Del	97 (72.4%)	319 (81.4%)	0.568	0.655 (0.153–2.789)
Allele Ins	37 (27.6%)	73 (18.6%)		
***UCP2*** **(*INDEL 45-bp*)**				
Del/Del	39 (58.2%)	88 (44.9%)	0.031 *	Del/Del vs. Others
Ins/Del	22 (32.8%)	90 (45.9%)		
Ins/Ins	6 (9.0%)	18 (9.2%)		2.031 (1.067–3.868)
Allele Del	100 (74.6%)	266 (67.9%)	0.617	1.367 (0.401–4.663)
Allele Ins	34 (25.4%)	126 (32.1%)		0.731 (0.214–2.495)

Ins: Insertion; Del: Deletion; OR: *odds ratio*; CI: confidence interval; ^a^. logistic regression adjusted for gender, age, and smoking. * *p*-value < 0.05.

## Data Availability

Not applicable.

## Supplementary Materials

The following supporting information can be downloaded at: https://www.mdpi.com/article/10.3390/genes14020461/s1, Table S1: SNP–SNP interaction models analyzed by the multifactorial dimensionality reduction (MDR) method, Figure S1: electropherogram of the five polymorphisms, Figure S2: electropherogram of the 61 markers of genomic ancestry, Figure S3: dendrogram (left) and the Fruchterman–Reingold graph (right) of the interactions between these SNPs.
