# Supplementary material for: Association between Polymorphism of Genes IL-1A, NFKB1, PAR1, TP53, and UCP2 and Susceptibility to Non-Small Cell Lung Cancer in the Brazilian Amazon
Source: Genes (Basel). 2023 Feb 10;14(2):461. doi: 10.3390/genes14020461 (PMC9957054; doi:10.3390/genes14020461)
Supplement: Supplementary file 1 [file genes-14-00461-s001.zip › genes-1938117-supplementary.docx.pdf]

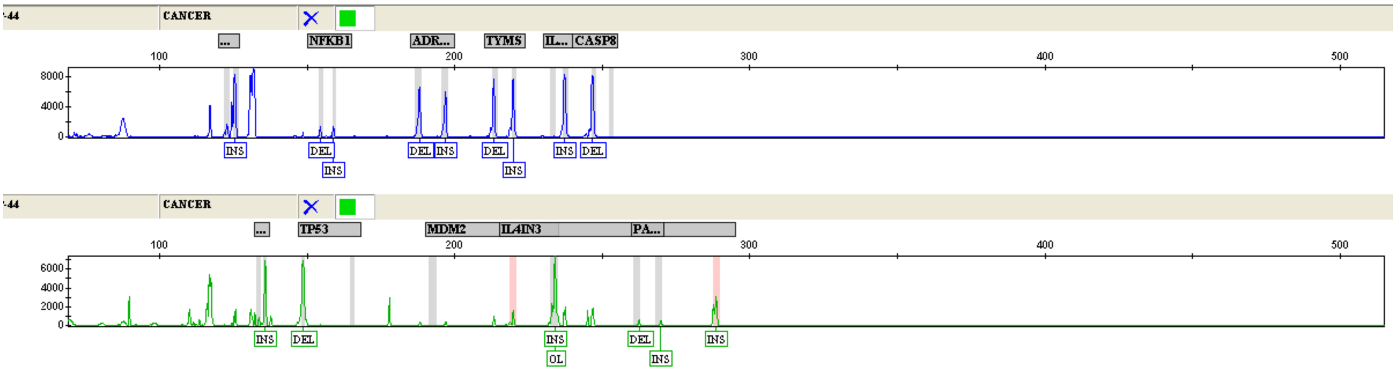

Figure S1. Electropherogram of the 5 polymorphisms. The figure represents an electropherogram interpreted by GeneMapper ID v. 3.2 software (Applied Biosystems, Life Technologies, Carlsbad, CA, USA). The gray rectangles have the name of the marker written (*NFKB1*, *TP53*, *PAR1*, *UCP2* and *IL-1A*) and delimit the range of known alleles. The peaks represent the alleles (INS or DEL). Each marker can have one peak (homozygous) or two peaks (heterozygous).

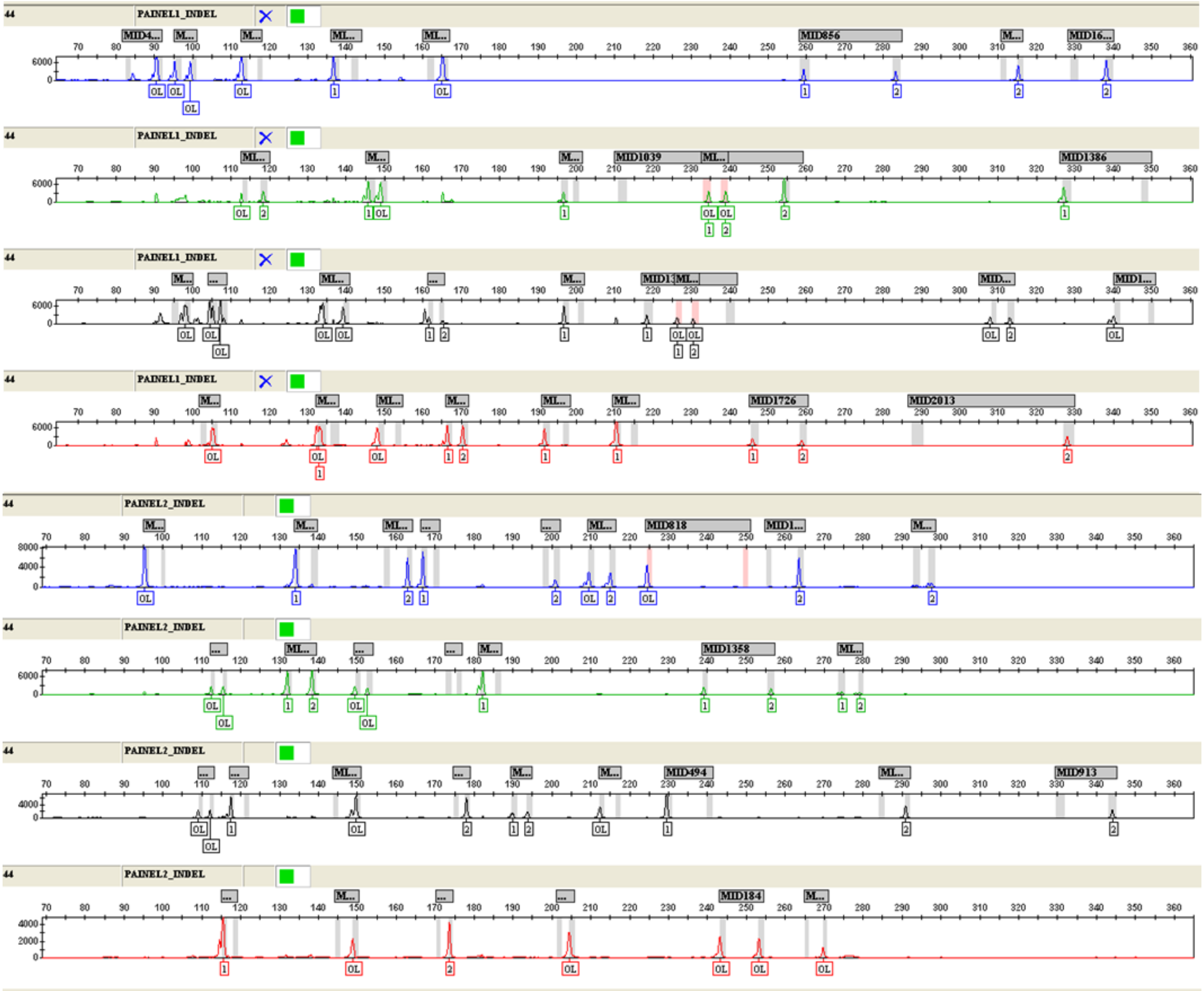

Figure S2. Electropherogram of the 61 markers of genomic ancestry. The figure represents an electropherogram interpreted by GeneMapper ID v. 3.2 software (Applied Biosystems, Life Technologies, Carlsbad, CA, USA). The gray rectangles have the name of the 61 markers written and delimit the range of known alleles. The peaks represent the alleles. Each marker can have one peak (homozygous) or two peaks (heterozygous).

The MDR was used to analyze the interaction between the five SNPs. The results of the MDR model analysis of SNP-SNP are shown in Table S1. The results considered that there was no significant influence between the investigated polymorphisms (p-value > 0.05). However, as shown in Figure S3, the dendrogram and the Fruchterman–Reingoldgraph describe this interaction between these SNPs. Entropy patterns recapitulate the main and/or interaction effect for each combination of attribute pairs. The strongest interaction effect was found between *PAR1* (rs11267092) and *IL-1A* (rs3783553), with information gain values of 2.11%. Furthermore, the second strongest effect was found between *TP53* (rs17878362) and *NFKB1* (rs28362491), with information gain values of 0.70%, and between *TP53* (rs17878362) and *UCP2* (INDEL 45-bp), with values of information gain of 0.50%.

Table S1. SNP–SNP interaction models analyzed by the multifactorial dimensionality reduction (MDR) method.

Model	Test Balance Accuracy	Cross-Validation Consistency	p-value[a]
UCP2	0.4363	6/10	0.7234
PAR1, IL1A	0.6619	10/10	0.3666
UCP2, PAR1, IL1A	0.5035	8/10	0.9841
UCP2, PAR1, IL1A, TP53	0.5180	9/10	0.9185
UCP2, PAR1, IL1A, TP53, NFKB1	0.4880	10/10	0.9458

UCP2 (INDEL 45-bp); *PAR1* (rs11267092); *IL1A* (rs3783553); *TP53* (rs17878362); *NFKB1* (rs28362491). a. Chi-square Test.

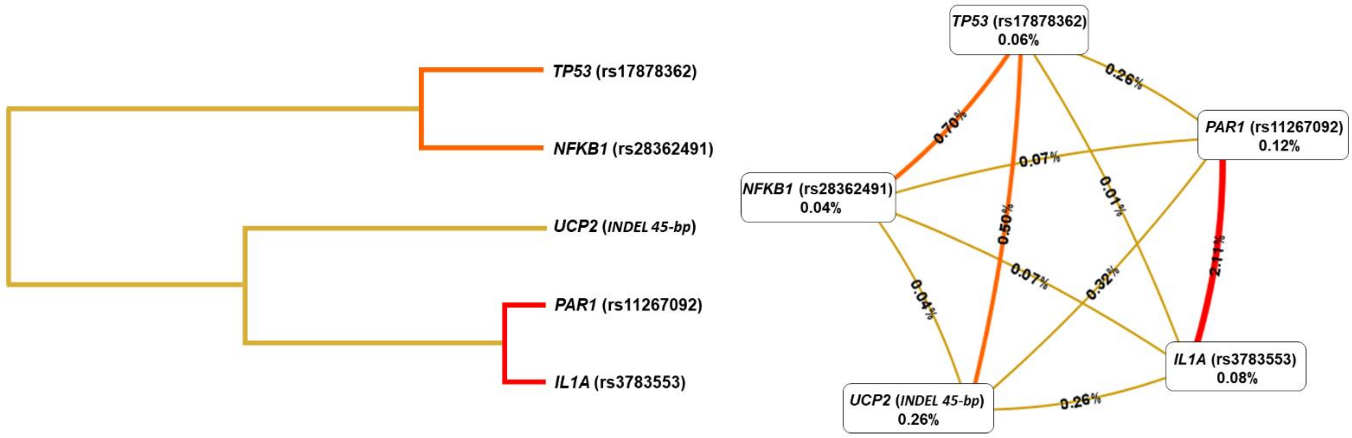

Figure S3. Dendrogram (left) and the Fruchterman-Reingold graph (right) for the interactions between these SNPs. Positive percent entropy indicates synergy. The orange and red lines indicated positive interaction, the others indicated weak interactions.
