# Supplementary figures and images for: Association between Polymorphism of Genes IL-1A, NFKB1, PAR1, TP53, and UCP2 and Susceptibility to Non-Small Cell Lung Cancer in the Brazilian Amazon
Source: Genes (Basel). 2023 Feb 10;14(2):461. doi: 10.3390/genes14020461 (PMC9957054; doi:10.3390/genes14020461)

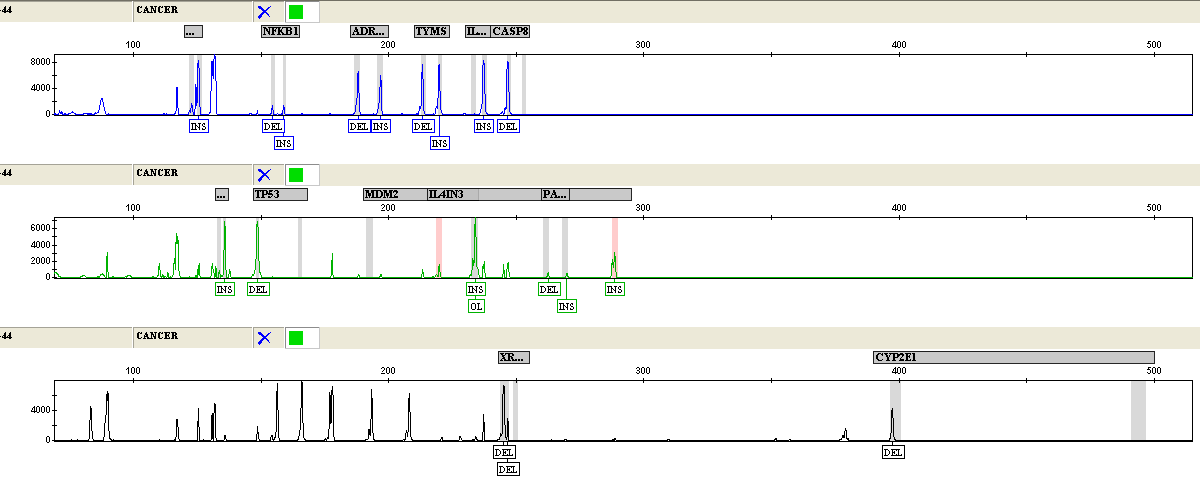

Supplement: Supplementary file 1 [file genes-14-00461-s001.zip › Figure S1.png]

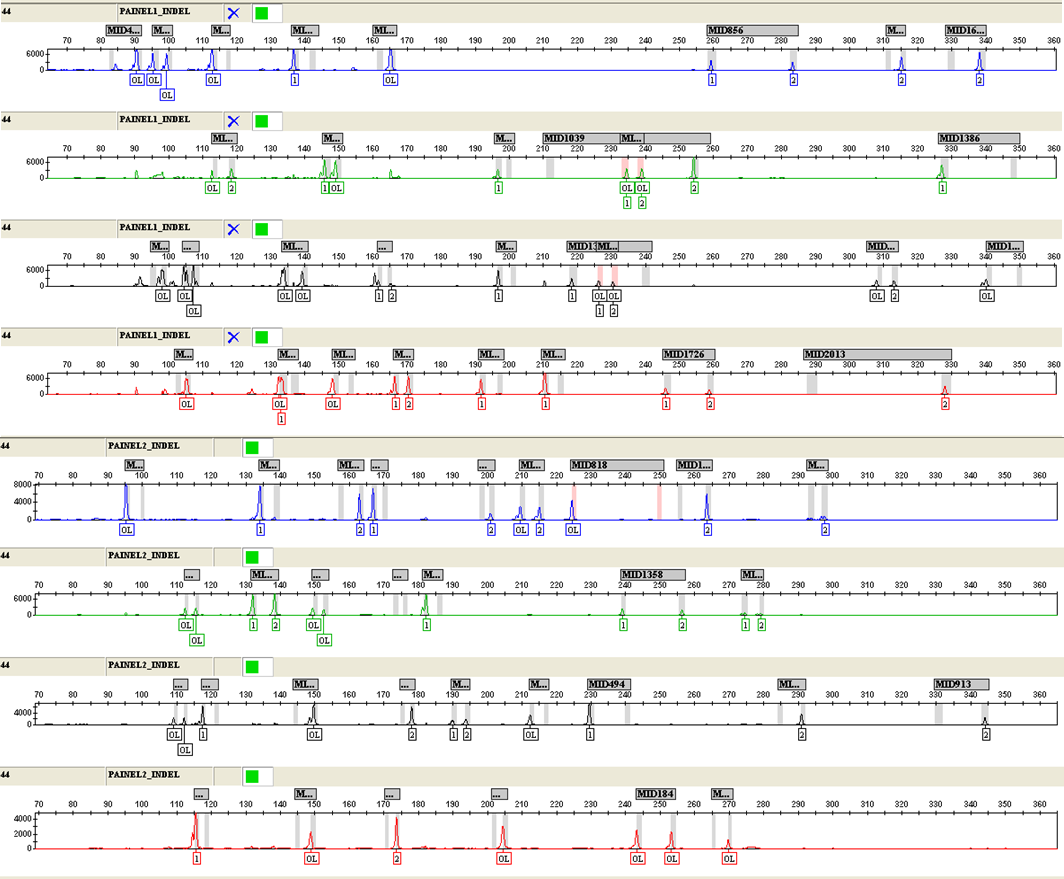

Supplement: Supplementary file 1 [file genes-14-00461-s001.zip › Figure S2.png]

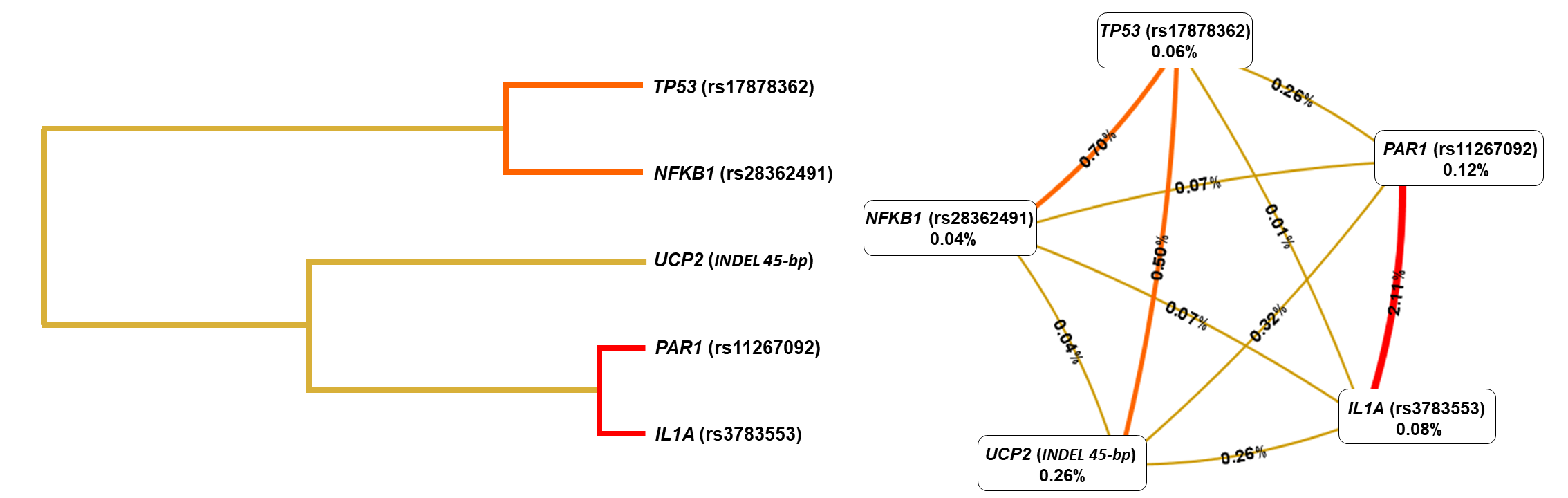

Supplement: Supplementary file 1 [file genes-14-00461-s001.zip › Figure S3.png]
